# Healthcare Utilization Following Implementation of a Pediatric Social Needs Screening Program

**DOI:** 10.1177/21501319261428865

**Published:** 2026-03-18

**Authors:** Ashley Gibson, Kaleigh Riggs-Harpur, Midhat Jafry, Mallika Mathur, Yen-Chi Le, Sandra McKay

**Affiliations:** 1 The University of Texas Health Science Center at Houston, USA

**Keywords:** social needs screening, social determinants of health, pediatric primary care, healthcare utilization, care coordination

## Abstract

**Objective:**

This study evaluated differences in healthcare utilization among a pediatric primary care population following implementation of a universal social needs screening and referral program. It was hypothesized that emergency, sick, and hospitalization visits would decrease post-implementation, with stable or increased preventive care.

**Methods:**

A retrospective, observational study was conducted using electronic health record data from 2 cohorts at different time periods at a large academic pediatric primary care clinic. The pre-implementation group included patients with a well-child visit from 08/2021 to 07/2022; the post-implementation group included patients from 09/2022 to 08/2023. Healthcare utilization measures of emergency department visits, hospitalizations, sick visits, and subsequent well-child visits were assessed from the index visit through the end of the group period. Differences in healthcare utilization were compared between cohorts using Mann-Whitney *U* (MW) tests, and associations of visit counts with social needs and care coordination referrals were examined using backwards-selected Zero-inflated Poisson regression models.

**Results:**

The study included 3889 pre-implementation and 3813 post-implementation patients. Post-implementation patients had fewer emergency visits (≥1 visit: 15.5% vs 13.1%; MW *P* = .002) and sick visits (≥2 visits: 29.3% vs 24.3%; MW *P* < .001). Well-child visits did not differ (MW *P* = .981). Hospitalization rates were 2.5% pre-implementation and 2.0% post-implementation (MW *P* = .120).

**Conclusions:**

Implementation of a universal social needs screening program was associated with improved patterns of pediatric healthcare utilization.

## Introduction

Social determinants of health (SDOH) are the conditions in which individuals live and grow that influence health outcomes, and these influences are particularly determinative during childhood, a period of significant mental and physical development.^1-5^ Unmet social needs during childhood are associated with a range of developmental problems, including physical and mental health conditions,^6-11^ increasing the risk of poor health across the life course.^12-14^ Social risk factors are also linked to increased healthcare utilization among pediatric patients,^15,16^ suggesting that addressing SDOH early may play a critical role in both improving health and optimizing healthcare use. In response, healthcare systems across the United States have increasingly adopted screening programs to identify and address SDOH in both adult and pediatric primary care settings. These programs often include referrals to care coordinators (eg, case managers or community health workers) to connect patients with community resources to address identified needs.^17^ The theoretical frameworks underpinning these interventions posit that screening for social needs and facilitating access to appropriate services can improve overall health.^18^

However, there is mixed evidence that social screening with resource navigation improves health and healthcare utilization. In adult populations, some studies have shown reduced Emergency Department (ED) visits and hospitalizations with SDOH screening and resource navigation,^19,20^ including the Accountable Health Communities study which has been the largest evaluation of a national SDOH screening program.^19^ However, other studies have found no benefit.^21^ A systematic review of 28 studies reported inconsistent effects on healthcare utilization.^22^ Fewer studies have been conducted in pediatric populations, and results have been similarly inconclusive. One randomized controlled trial (RCT) found in a secondary analysis that caregiver connection to community resources was associated with lower hospitalization rates in children,^23^ while a retrospective study linked social risk screening to increased attendance at recommended well-child visits.^24^ However, neither study found significant reductions in ED visits.^23,24^ In contrast, an RCT involving infants connected to a family specialist did find a reduction in ED utilization.^25^ Given the long-term health implications established early in life, these findings underscore a critical need for further research to clarify the impact of SDOH screening on healthcare utilization in pediatric primary care, where the potential for long-term benefits is substantial but evidence remains limited.

The objective of this study was to evaluate healthcare utilization among pediatric patients after implementation of a primary care SDOH screening program. Specifically, the study had 2 aims: (1) to evaluate the difference in healthcare utilization between pediatric patients seen prior to and following implementation and (2) to evaluate the relationship of SDOH needs and referrals with healthcare utilization. We hypothesized that healthcare utilization would be associated with social needs identification and referrals and that there would be a decrease of illness-related visits during the post implementation period, including a decrease of Emergency Department visits, hospitalizations, and sick visits, while routine care visits would be unchanged or increased. This hypothesis is grounded in the idea that social needs may influence both health and healthcare-seeking behavior, thereby shaping patterns of illness-related care.

## Methods

### Study Design and Population

This retrospective observational study used electronic health record (EHR) data from 2 cohorts at distinct time periods in a large academic pediatric primary care clinic serving a diverse urban population in Houston, Texas. The first time period represented the pre-implementation period of the SDOH screening and referral program, and the second represented the post-implementation period. Beginning in August 2022, the clinic implemented a universal SDOH screening and resource referral program to address unmet social needs among pediatric patients.

As part of the SDOH program, caregivers of all children presenting for a routine preventive visit, called a well-child visit (WCV) were asked to complete a 12-item SDOH screening questionnaire. The screening questionnaire was integrated into the electronic health record (ie, Epic EHR system). The pediatric SDOH screening questionnaire included 12 questions across 6 SDOH domains: food insecurity, transportation needs, housing instability, financial resource strain, health literacy, and medical-legal needs (also referred to as health-harming legal needs). These SDOH domains were selected based on a systematic literature review, priority areas from Healthy People 2030, review of validated questions, and ability of staff to address the domains.^26^ For families who screened positive for social needs, physicians could recommend community resources and refer patients to a social worker, community health worker (CHW), or care manager.^26^ Referral to a social worker, CHW, or care manager was dependent upon the SDOH need the patient flagged positive for. Medical-legal needs were directed to clinic social workers, while the other SDOH needs from the screening questionnaire were directed to CHWs. Physicians continued to have the ability to refer to care coordination providers as needed for additional needs that were identified during the visit (eg, social work referral for behavioral health needs and care management for medical care coordination). Prior to program implementation in August 2022, physicians had the ability to refer to these care coordination providers as needed, but the program introduced systematic changes for universal SDOH screening at pediatric WCV. As reported in a prior publication evaluating the first 4 months of this program, 32% of patients screened positive for at least 1 SDOH domain, with financial resource strain (16.5%), housing instability (14.1%), and food insecurity (12.8%) as the most prevalent concerns.^26^

This study included all patients aged 0 to 21 years who came to the pediatric clinic for a WCV. The pre-implementation cohort comprised patients seen for a WCV between August 1, 2021, and July 31, 2022, and the post-implementation cohort included patients seen for a WCV between September 1, 2022, and August 31, 2023. Visits during August 2022 were excluded due to the program transition phase. The index WCV during the respective group period (pre-implementation or post-implementation) was considered the exposure in this study. Some patients had a WCV in both time periods and were therefore included in both cohorts, as inclusion was based solely on the time period in which care was received. This time-defined approach reflects a cohort structure in which groups are formed based on exposure period, rather than requiring the same patients to be present in both groups. The study was approved by the Institutional Review Board at the affiliated institution (HSC-MS-23-0862).

### Study Measures

Data for this study was obtained electronically from the EHR. Data on patient demographics, SDOH needs identified from the screening questionnaire, and referrals to care coordination (CHW, Social Work, or Care Management) were obtained from the patients’ index WCV during their respective group period. Healthcare utilization outcomes included 4 prespecified categories: Emergency Department visits, inpatient hospitalizations, sick visits, and well-child visits. Encounters in these categories were included as outcomes if they were documented in the EHR after the time of the index WCV through the end of the group period (July 31, 2022 for the pre-implementation group and August 31, 2023 for the post-implementation group). These encounters were captured across multiple hospitals and outpatient clinics that are affiliated with the academic institution. While all 4 outcomes were analyzed to understand the broader utilization profile, ED visits were identified as the primary outcome for reporting purposes because they are frequently evaluated in prior SDOH research and are considered particularly sensitive to access barriers and unmet social needs. Encounters were classified as well-child visits if this was indicated by 2 or more of the following EHR fields: visit reason, diagnosis codes, and billing codes. All other outpatient visits were categorized as sick visits, which included visits for acute illness and follow-up care for chronic conditions, such as specialist visits. These visits were analyzed together to describe illness-related outpatient care, in contrast to preventive well-child care. Outpatient visits included both office and telemedicine encounters, excluding visits that were cancelled or no-showed. Because patients varied in the amount of time they were observed after the index WCV, varying length of patient study time was controlled for in the regression analysis. Length of study time was defined as the number of days between each patient’s index WCV and the end of the applicable group period.

### Data Analysis

Descriptive statistics with frequencies, percentages, and medians with interquartile ranges (IQRs) were reported. Chi-square test was used to assess for differences between the cohorts for sex, race, ethnicity, language spoken at home, and referrals to care coordination. Mann-Whitney U test was used to determine whether the median age differed between the 2 groups. Mann-Whitney U was used to evaluate the first aim, assessing for differences in healthcare utilization between the pre- and post-implementation groups. In a post-hoc analysis, we further assessed paired Wilcoxon Rank tests for the subset of patients present in both cohorts to compare outcomes. The number of well-child visits, sick visits, ED visits, and hospitalizations were evaluated, as well as the rates of these visits (number of visits divided by length of study).

For the second aim, multiple Zero-inflated Poisson (ZIP) regressions were used to evaluate the association between healthcare visits with SDOH needs and care coordination referrals. ZIP regression models visit counts by estimating 2 related components. Because there is an excess of people with zero visits, an odds ratio for zero visits is given. Meanwhile, among those who have a non-zero number of visits an incident risk ratio is estimated relating the number of visits. Separate ZIP models were fit for each healthcare utilization outcome (dependent variables): number of well-child visits, sick visits, ED visits, and hospitalizations. Independent variables included SDOH needs and care coordination referrals, along with length of study time. The ZIP regressions included SDOH needs in 2 different ways. The first set of models included SDOH as 11 variables from separate questions of the questionnaire, with food insecurity included as a single variable from the validated 2-question screen (Supplement). The second set of models included SDOH as 6 domains. For each outcome, an initial full model was specified, and variables were reduced using backward selection to retain factors that were statistically significant or acted as confounders (ie, meaningfully altering other coefficients when removed). Length of study time was included as a covariate in all models. A *P*-value below .05 was considered statistically significant. All statistical analyses were performed using R 4.5.0 Statistical software.

## Results

### Comparison of Patient Characteristics and Referrals

A total of 3889 patients were included in the pre-implementation group and 3813 patients were included in the post-implementation group. Because each group represented a distinct 12-month period, the total number of patients differed slightly between the pre- and post-implementation cohorts. Of these patients, 1928 (33.4%) had a well-child visit in both periods and were therefore included in both cohorts, while 3846 (66.6%) were included in only 1 cohort. As shown in Table 1, the median age was 4 years (IQR = 1-10) in the pre-implementation group and 4 years (IQR = 0-10) in the post-implementation group. In both the pre- and post-implementation groups, the majority of patients were male (52.4%; 53.6%), English-speaking (92.6%; 91.4%), and documented non-Hispanic ethnicity (63.7%; 62.1%). There were no significant differences in age, sex, language, or ethnicity. There was a significant difference in patient race (*P* < .001). In the pre-implementation group, compared to post-implementation, the race classification of Black or African American was more common (53.9%; 44.4%), while the race classification of Other (25.7%; 29.1%) and unknown documentation for race (11.4%; 18.7%) were more common post-implementation.

**Table 1.** Demographic Characteristics and Referrals to Care Coordination for Patients Before and After Implementation of the Social Needs Screening Program.

	Pre-implementation group	Post-implementation group	*P*-value
Characteristic	N (%)	N (%)	
Total patients	3889	3813	
Age in years at initial WCV (median, IQR)	4 (1, 10)	4 (0, 10)	.197
Sex			
Female	1851 (47.6)	1768 (46.4)	.280
Male	2038 (52.4)	2045 (53.6)	
Primary language spoken at home			
Arabic	11 (0.3)	14 (0.4)	.795
English	3603 (92.6)	3486 (91.4)	
Sign Language	12 (0.3)	18 (0.5)	
Spanish	217 (5.6)	243 (6.4)	
Other	22 (0.6)	33 (0.9)	
Unknown	24 (0.6)	19 (0.5)	
Ethnicity			
Hispanic	1169 (30.1)	1221 (32.0)	.282
Not Hispanic	2479 (63.7)	2368 (62.1)	
Unknown	241 (6.2)	224 (5.9)	
Race			
Native American or Alaska Native	4 (0.1)	3 (0.1)	<.001*
Asian	34 (0.9)	33 (0.9)	
Black or African American	2097 (53.9)	1692 (44.4)	
White	312 (8.0)	263 (6.9)	
Other	998 (25.7)	1110 (29.1)	
Unknown	444 (11.4)	712 (18.7)	
Referral to CHW			
Not referred	3878 (99.7)	3148 (82.6)	<.001*
Referred	11 (0.3)	665 (17.4)	
Referral to social work			
Not referred	3675 (94.5)	3540 (92.8)	.003*
Referred	214 (5.5)	273 (7.2)	
Referral to care management			
Not referred	3836 (98.6)	3744 (98.2)	.116
Referred	53 (1.4)	69 (1.8)	

Abbreviations: CHW, community health worker; IQR, interquartile range; WCV, Well child visit.

* Significant association, *P* < .05.

Table 1 also shows the referrals for care coordination in both the pre- and post-implementation groups. In the post-implementation group, there were significantly more referrals to Community Health Workers (*P* < .001) and Social Workers (*P* = .003). Eleven patients (0.3%) were referred to CHWs pre-implementation compared to 665 (17.4%) post-implementation, while 214 (5.5%) were referred to Social Work pre-implementation compared to 273 (7.2%) post-implementation. The number of patients referred to Care Management was 53 (1.4%) pre-implementation compared to 69 (1.8%) post-implementation, which was not a statistically significant difference (*P* = .116).

### Comparison of Healthcare Utilization

The distribution of ED visits differed significantly between groups, with more ED visits in the pre-implementation group (*P* = .002), as shown in Table 2. 15.5% of patients had at least 1 ED visit pre-implementation, compared to 13.1% post-implementation. Hospitalizations showed a similar trend with 2.5% of patients having a hospitalization pre-implementation compared to 2.0% post-hospitalization, although this difference was not statistically significant (*P* = .120). Patients in the post-implementation period were more likely to have 0 or 1 sick visits and less likely to have 2 or more sick visits (*P* < .001). In the pre-implementation period, 29.3% had 2 or more sick visits, while 24.3% in the post-implementation period had 2 or more sick visits. The number of well-child visits did not differ between the pre- and post-implementation periods (*P* = .981). In both groups, the majority of patients had 0 well-child visits after their index WCV (65.8%; 66.0%). Analysis of the rates of ED, hospitalization, sick, and well-child visits showed the same results as the analysis of number of visits.

**Table 2.** Healthcare Visits Among Cohorts Before and After Implementation of the Social Needs Screening Program.

	Pre-implementation group	Post-implementation Group	*P*-value
Visit count	N (%)	N (%)	
Total patients	3889	3813	
Well child visits			
0	2558 (65.78)	2515 (65.96)	.981
1	647 (16.64)	598 (15.68)	
2-4	631 (16.23)	643 (16.86)	
5+	53 (1.36)	57 (1.59)	
Sick visits			
0	2024 (52.04)	2121 (55.63)	<.001*
1	725 (18.64)	764 (20.04)	
2-4	753 (19.36)	685 (17.96)	
5+	387 (9.95)	243 (6.37)	
Emergency department visits			
0	3286 (84.49)	3313 (86.89)	.002*
1	408 (10.49)	361 (9.47)	
2-4	179 (4.60)	131 (3.44)	
5+	16 (0.41)	8 (0.21)	
Hospitalizations			
0	3791 (97.48)	3737 (98.01)	.120
1	62 (1.59)	49 (1.29)	
2-4	30 (0.77)	21 (0.55)	
5+	6 (0.15)	6 (0.16)	

* Significant association, *P* < .05.

In the analysis restricted to the 1928 patients with a WCV in both pre-implementation and post-implementation periods, the patients had fewer ED visits (*P* < .001) and fewer sick visits (*P* < .001) post-implementation. These patients also had fewer WCV in the post-implementation period (*P* < .001). The results of the paired analysis comparing the number of visits are shown in Table 3. Analysis of the rates of visits showed the same results as the analysis of number of visits. The median age increased in the post-implementation period as expected for the paired analysis, with the median age 4 years (IQR = 1-9) pre-implementation and 5 years (IQR = 2-10) post-implementation.

**Table 3.** Healthcare Visits Before and After Implementation of the Social Needs Screening Program Among Patients Seen in Both Periods (N = 1928).

Visit count	Pre-implementation N (%)	Post-implementation N (%)	*P*-value
Well child visits			
0	1178 (61.10)	1413 (73.29)	<.001*
1	313 (16.23)	285 (14.78)	
2-4	398 (20.64)	230 (11.93)	
5+	39 (2.02)	0 (0)	
Sick visits			
0	886 (45.95)	1034 (53.63)	<.001*
1	359 (18.62)	391 (20.28)	
2-4	440 (22.82)	362 (18.78)	
5+	243 (12.60)	141 (7.31)	
Emergency department visits			
0	1589 (82.42)	1665 (86.36)	<.001*
1	229 (11.88)	189 (9.80)	
2-4	100 (5.19)	68 (3.53)	
5+	10 (0.52)	6 (0.31)	
Hospitalizations			
0	1866 (96.78)	1877 (97.35)	.060
1	43 (2.23)	37 (1.92)	
2-4	16 (0.83)	11 (0.57)	
5+	3 (0.16)	3 (0.16)	

* Significant association, *P* < .05.

### Relationship of Social Needs and Referrals With Healthcare Utilization

Both components of the Zero-inflated Poisson models (odds ratios for zero visits and incident risk ratios for visit counts) are reported in Tables 4 to 7. Referrals to a CHW were associated with fewer WCV in both regression models including SDOH as single variables and combined domains (Incident risk ratio (IRR) = 0.79, *P* = .003; IRR = 0.80, *P* = .005), as shown in Table 4. When medical-legal needs were included as single variables, a concern about custody, guardianship, or child support was associated with fewer WCV (IRR = 0.41, *P* < .001), while a caregiver reporting legal issues they wished to discuss with an attorney was associated with increased WCV (IRR = 1.63, *P* = .014). The combined domain of medical-legal needs was not significantly associated with WCV.

**Table 4.** Final Regression Model for outcome of Well Child Visits.

	SDOH as single variables			SDOH combined domains		
Variable	IRR	95% CI	*P*-value	IRR	95% CI	*P*-value
Study time	1.01	1.00, 1.01	<.001*	1.01	1.00, 1.01	<.001*
Transportation needs	0.84	0.66, 1.06	.143	0.83	0.66, 1.04	.102
Financial resource strain	1.08	0.91, 1.27	.396	1.07	0.91, 1.26	.431
Medical-legal needs						
Child separation due to foster care/immigration	0.46	0.16, 1.35	.158			
Concerns about custody, guardianship, or child support	0.41	0.25, 0.68	<.001*			
Legal issues to discuss with an attorney	1.63	1.10, 2.40	.014*			
Concerns about losing a job						
Referral to CHW	0.79	0.68, 0.93	.003*	0.80	0.69, 0.94	.005*
Referral to social work	1.20	0.96, 1.50	.104			
	OR	95% CI	*P*-value	OR	95% CI	*P*-value
Zeroes	0.73	0.62, 0.85	<.001*	0.74	0.62, 0.86	<.001*

Abbreviations: CHW, Community Health Worker; CI, confidence interval; IRR, incident risk ratio; OR, odds ratio; SDOH, social determinants of health.

* Significant association, *P* < .05.

**Table 5.** Final Regression Model for outcome of Sick Visits.

	SDOH as single variables			SDOH combined domains		
Variable	IRR	95% CI	*P*-value	IRR	95% CI	*P*-value
Study time	1.00	1.00, 1.00	<.001*	1.00	1.00, 1.00	<.001*
Low health literacy	0.61	0.42, 0.89	.011*	0.62	0.42, 0.90	.012*
Food insecurity	1.27	1.10, 1.47	.001*	1.25	1.09, 1.45	.002*
Housing instability				1.15	1.01, 1.32	.036*
Transportation needs				0.89	0.75, 1.07	.217
Financial resource strain	0.99	0.87, 1.13	.910	0.97	0.85, 1.11	.649
Medical-legal needs				0.80	0.65, 0.99	.036*
Child separation due to foster care/immigration	0.33	0.11, 0.99	.048*			
Concerns about custody, guardianship, or child support	0.60	0.42, 0.86	.005*			
Legal issues to discuss with an attorney	1.13	0.83, 1.55	.426			
Concerns about losing a job	0.82	0.61, 1.10	.176			
Referral to Social Work	1.23	1.04, 1.45	.015*	1.17	0.99, 1.39	.059
Referral to Care Management	1.98	1.61, 2.44	<.001*	2.04	1.67, 2.49	<.001*
	OR	95% CI	*P*-value	OR	95% CI	*P*-value
Zeroes	0.77	0.68, 0.87	<.001*	0.77	0.67, 0.86	<.001*

Abbreviations: CI, confidence interval; IRR, incident risk ratio; OR, odds ratio; SDOH, social determinants of health.

* Significant association, *P* < .05.

**Table 6.** Final Regression Model for outcome of Emergency Department Visits.

	SDOH as single variables			SDOH combined domains		
Variable	IRR	95% CI	*P*-value	IRR	95% CI	*P*-value
Study time	1.01	1.00, 1.01	<.001*	1.01	1.00, 1.01	<.001*
Low health literacy				0.65	0.26, 1.66	.370
Housing instability				1.37	1.00, 1.88	.054
Financial resource strain				1.32	0.99, 1.78	.063
Medical-legal needs				0.52	0.30, 0.88	.016*
Child separation due to foster care/immigration						
Concerns about custody, guardianship, or child support						
Legal issues to discuss with an attorney						
Concerns about losing a job						
Referral to Social Work				1.37	0.93, 2.03	.112
	OR	95% CI	*P*-value	OR	95% CI	*P*-value
Zeroes	2.38	2.09, 2.68	<.001*	2.06	1.50, 2.61	<.001*

Abbreviations: CI, confidence interval; IRR, incident risk ratio; OR, odds ratio; SDOH, social determinants of health.

* Significant association, *P* < .05.

**Table 7.** Final Regression Model for Outcome of Hospitalizations.

	SDOH as single variables			SDOH combined domains		
Variable	IRR	95% CI	*P*-value	IRR	95% CI	*P*-value
Study time	1.00	1.00, 1.01	.050*	1.00	1.00, 1.01	.086
Housing instability				4.87	1.74, 13.70	.003*
Transportation needs	1.64	0.64, 4.20	.299	1.50	0.57, 3.91	.408
Financial resource strain	0.73	0.33, 1.62	.435	0.25	0.09, 0.67	.006*
Medical-legal needs				0.19	0.05, 0.75	.018*
Child separation due to foster care/immigration						
Concerns about custody, guardianship, or child support						
Legal issues to discuss with an attorney						
Concerns about losing a job						
Referral to CHW	1.72	0.81, 3.68	.159	1.30	0.63, 2.68	.480
Referral to Social Work	2.03	0.93, 4.41	.074	2.18	0.95, 4.98	.064
	OR	95% CI	*P*-value	OR	95% CI	*P*-value
Zeroes	25.77	12.34, 41.95	<.001*	27.15	13.02, 48.28	<.001*

Abbreviations: CHW, Community Health Worker; CI, confidence interval; IRR, incident risk ratio; OR, odds ratio; SDOH, social determinants of health.

* Significant association, *P* < .05.

Referrals to Care Management were associated with a higher number of sick visits in both regression models including SDOH as single variables and combined domains (IRR = 1.98, *P* < .001; IRR = 2.04, *P* < .001; Table 5). Referrals to Social Work were also associated with more sick visits in the model with individual SDOH variables (IRR = 1.23, *P* = .015), though the association was not statistically significant with combined SDOH domains. The SDOH need of food insecurity was consistently associated with more sick visits across both models (IRR = 1.27, *P* = .001; IRR = 1.25, *P* = .002), and housing instability was associated with more sick visits in the model with combined domains (IRR = 1.15, *P* = .036). In contrast, lower numbers of sick visits were observed for patients reporting low health literacy in both models (IRR = 0.61, *P* = .011; IRR = 0.62, *P* = .012), and for child separation due to foster care or immigration (IRR = 0.33, *P* = .048) and a concern about custody, guardianship, or child support (IRR = 0.60, *P* = .005) in the model with individual SDOH variables. The combined domain of medical-legal needs was also associated with fewer sick visits (IRR = 0.80, *P* = .036).

The combined domain of medical-legal needs was associated with fewer ED visits (IRR = 0.52, *P* = .016) and fewer hospitalizations (IRR = 0.19, *P* = .018). Table 6 shows the regression results for ED visits, while the results for hospitalizations are shown in Table 7. Financial resource strain also showed an association with fewer hospitalizations (IRR = 0.25, *P* = .006), while housing instability was associated with more hospitalizations (IRR = 4.87, *P* = .003). No referrals for care coordination were associated with ED visits or hospitalizations in the adjusted analyses. Length of study time was positively associated with the number of visits for WCV, sick visits, ED visits, and hospitalizations.

## Discussion

This study is among a limited number that have examined healthcare utilization following the implementation of a primary care-based SDOH screening and referral program in a pediatric population. The primary outcome, ED visits, decreased in the post-implementation period, suggesting a potential shift away from acute, unscheduled care. Patients in the post-implementation period also had fewer sick visits, while the number of well-child visits remained unchanged in the unpaired analysis. Taken together, these findings suggest a more favorable pattern of healthcare utilization, with patients utilizing primary care preventive services but not requiring illness-related visits as frequently. Additionally, hospitalizations remained stable across periods, indicating that the decrease in outpatient illness visits was not due to worsening clinical severity that would have resulted in more inpatient care.

Analysis of the subset of patients with a WCV in both time periods showed similar results with a decrease in ED visits, a decrease in sick visits, and stable hospitalizations. Unlike the unpaired cohort analysis, the paired analysis showed significantly fewer WCV in the post-implementation period. However, this is likely due to the increased age of these patients in the later period, as the recommended number of WCV decreases as children age. For example, more WCV are recommended in the first year of life, followed by progressively fewer visits in early childhood and annual visits thereafter. In the unpaired analysis, age distributions were similar between the pre- and post-implementation groups, suggesting comparable opportunities for additional well-child visits during follow-up.

Although there was improved healthcare utilization during the post-implementation period as hypothesized, the relationship between SDOH needs identification and referrals with healthcare utilization in the regression analysis was complex. Referrals to CHWs were associated with fewer well-child visits, while referrals to Care Management and Social Work were associated with more sick visits. These patterns likely reflect the clinical complexity of referred patients rather than a causal effect of the referrals, as WCVs were unchanged and sick visits decreased in the post-implementation period with an increased number of referrals. Similarly, the relationship between SDOH needs identified with screening and healthcare utilization was not straightforward. Some SDOH domains such as food insecurity and housing instability were linked to increased utilization. However, medical-legal needs were associated with decreased utilization, which could reflect systemic or other unmeasured barriers to seeking care. These findings should be interpreted as associations, recognizing that unmeasured confounding and other patient or context-related factors may have contributed to the observed patterns.

The results of this study are consistent with prior research showing improvements in healthcare utilization following SDOH interventions, even when improvement in social needs has been limited or inconsistent. The nationwide study evaluating the Accountable Health Communities model over 5 years reported no significant differences in utilization of community services regardless of assistance offered. However, the report did highlight a reduction in ED visits, particularly for nonemergent visits, and hospitalized stays.^19^ Another study among adults with high healthcare utilization found a reduced likelihood of utilization, including visits inpatient, outpatient, and to the ED, following a telephone-based social needs screening, although the authors noted that only a minority of those with a need were able to connect with resources to address the needs.^20^ These recent insights have shaped a revision of the original logic model, shifting the focus from a linear pathway (eg, SDOH screening to community resource connections to improved outcomes) to a more multifactorial concept of how efforts involving social factors influence care.^27^ Namely, navigating SDOH may involve both resource connection and improved interactions with the healthcare system (eg, building provider-patient trust, tailoring clinical care). Moreover, a growing body of research corroborates the association between better emotional support from social connections and reduced healthcare utilization.^27-30^

Still, resource connection may be an important factor, particularly for some patients. In the Accountable Health Communities study, some groups of patients including underserved racial and ethnic groups and patients with multiple social needs were more likely to have their needs resolved.^19^ Other studies have found an overall improvement in community resource connection and resolution of social needs. For instance, a cluster RCT conducted in 8 urban community health centers showed screening and referral among infants’ mothers increased enrollment in new community resources,^31^ while another RCT among caregivers of children in 2 urban hospitals found that patient navigators reduced reported social needs at 4-month follow ups and improved the caregivers’ report of their child’s overall health.^32^ In a follow-up analysis of patients from the first 4 months of the SDOH program in this current study, 75% of those re-screened within 1 year reported improvement or resolution of their social needs. While most patients reported improvement regardless of referral status, those referred to care coordination were more likely to report improvement.^33^

The current study has a few important limitations. First, because this was an observational cohort comparison of patients from 2 different time periods, interpretation of causality is limited, as differences in healthcare utilization could have occurred due to temporal changes unrelated to the program. Still, the findings align with prior research demonstrating improved outpatient and acute care utilization with implementation of SDOH programs. Another important limitation is SDOH screening occurred as part of the program, thus documentation of SDOH needs was not available in the pre-implementation group. Therefore, differences in SDOH needs between the 2 cohorts were not assessed, and the associations of SDOH needs with referrals and healthcare utilization were limited to the post-implementation group.

Additional limitations resulted from the availability of retrospective EHR data. For example, outpatient visits were classified as WCVs versus sick visits based on the available EHR information, and misclassification from incomplete data is therefore possible. Because the retrospective EHR data did not reliably distinguish between acute illness visits and follow-up care for chronic conditions, these encounters were analyzed together as sick visits, which may have limited the ability to detect more nuanced patterns in outpatient illness-related utilization. A related limitation concerns patient demographic information. More patients had documented race classifications of Other and Unknown race in the post-implementation period, which could reflect population changes but could also be from changes in documentation practices. Additionally, the analysis only included encounters in the EHR system, and encounters at outside institutions utilizing a different EHR could have been missed.

Despite its limitations, this study provides valuable evidence on healthcare utilization patterns following the implementation of a primary care-based SDOH screening and referral program in a pediatric population. By including all patients seen in a large primary care clinic, not just those with identified social needs, this analysis evaluates the effects of a universal screening approach across the entire patient population. Even with this broad inclusion, significant changes in healthcare utilization were observed. Future research is needed to determine whether these changes reflect improved patient health, more efficient use of healthcare services (eg, reduction of ED visits for non-emergencies), or a combination of both. Optimizing healthcare utilization is important for enhancing patient outcomes and experiences, as well as for promoting efficient and effective use of healthcare system resources.

## Conclusion

This study contributes to the growing evidence base on the impact of SDOH screening in pediatric primary care by demonstrating changes in healthcare utilization following program implementation. Patients in the post-implementation period had fewer ED visits and fewer sick visits, while the numbers of well-child visits did not change, suggesting an overall improved pattern of healthcare utilization. However, the complex relationships observed between social needs, referrals, and utilization underscore the need for further research to clarify causal pathways and identify which components of SDOH interventions are most effective. Prospective studies are needed that assess long-term health outcomes, healthcare use, and patient-centered measures including social needs, to more fully evaluate the effects of SDOH screening programs on pediatric care.

## Supplemental Material

sj-docx-1-jpc-10.1177_21501319261428865 – Supplemental material for Healthcare Utilization Following Implementation of a Pediatric Social Needs Screening Program Supplemental material, sj-docx-1-jpc-10.1177_21501319261428865 for Healthcare Utilization Following Implementation of a Pediatric Social Needs Screening Program by Ashley Gibson, Kaleigh Riggs-Harpur, Midhat Jafry, Mallika Mathur, Yen-Chi Le and Sandra McKay in Journal of Primary Care & Community Health
