# Supplementary material for: Healthcare Utilization Following Implementation of a Pediatric Social Needs Screening Program
Source: J Prim Care Community Health. 2026 Mar 18;17:21501319261428865. doi: 10.1177/21501319261428865 (PMC13009944; doi:10.1177/21501319261428865)
Supplement: sj-docx-1-jpc-10.1177_21501319261428865 – Supplemental material for Healthcare Utilization Following Implementation of a Pediatric Social Needs Screening Program [file sj-docx-1-jpc-10.1177_21501319261428865.docx]

| **Supplement. Social Determinants of Health (SDOH) Screening Questions and Domains**^26^ | | | |
| --- | --- | --- | --- |
| **SDOH Domain** | **Question** | **Answer Options** | **Source** |
| **Health Literacy** | 1. How confident are you filling out medical forms by yourself? | 5-point Likert scale:  Extremely confident − Not at all | Chew et al.^34^ |
| **Transportation Needs** | 2. In the past 12 months, has lack of transportation kept you from medical appointments or from getting medications? | Yes/No | Adapted from the Protocol for Responding to and Assessing Patients’ Assets, Risks, and Experiences (PRAPARE).^35^ |
| **Food Insecurity** | 3a. Within the past 12 months, you worried that your food would run out before you got the money to buy more. | 3-point Likert scale:  Never true − Often true | Children’s Health Watch The Hunger Vital Sign^TM^.^36^ |
|  | 3b. Within the past 12 months, the food you bought just didn’t last and you didn’t have money to get more. | 3-point Likert scale:  Never true − Often true |  |
| **Housing Instability** | 4. In the last 12 months, was there a time when you were not able to pay the mortgage or rent on time? | Yes/No | Children’s Health Watch Housing Stability Vital Sign^TM^.^37^ |
|  | 5. In the last 12 months, how many places have you lived? | 0−2, 3 or greater |  |
|  | 6. In the last 12 months, was there a time when you did not have a steady place to sleep or slept in a shelter (including now)? | Yes/No |  |
| **Financial Resource Strain** | 7. How hard is it for you to pay for the very basics like food, housing, medical care, and heating? | 5-point Likert scale:  Very hard − Not hard at all | Puterman et al.^38^ |
| **Medical-Legal Needs** | 8. Has your child ever been separated due to foster care or immigration? | Yes/No | Adapted from the National Center for Medical-Legal Partnership.^39^ |
|  | 9. Do you have concerns about custody, guardianship or child support? | Yes/No |  |
|  | 10. Do you have any legal issues in which you would like to talk to an attorney? | Yes/No |  |
|  | 11. Does anyone in your house have concerns about losing a job or being at risk for losing a job? | Yes/No |  |
